# Judgments about appropriate foods for infants: Associations with parents’ own food preferences

**DOI:** 10.3389/fnut.2022.954981

**Published:** 2022-08-18

**Authors:** Jasmine M. DeJesus

**Affiliations:** Department of Psychology, University of North Carolina at Greensboro, Greensboro, NC, United States

**Keywords:** cognitive bias, feeding practices, food selection, infant feeding, food beliefs

## Abstract

When infants begin to eat solid foods (recommended at around 6 months of age), parents have a huge variety of choices in terms of what foods to offer. The present studies examine parents’ judgments about foods for infants. Participants included parents recruited from Prolific (*n* = 99), who were shown descriptions of foods offered to infants (including familiar and unfamiliar foods at 6-, 9-, and 12-months) and a set of control foods eaten by adults. Participants rated each food based on how appropriate they thought it was for an infant and how much they personally wanted to eat the food. Parents rated foods as more appropriate for infants if they were familiar (vs. unfamiliar) and offered to younger infants (6- vs. 12-month-olds, or infant foods vs. adult foods), but demonstrated the opposite pattern when considering whether they wanted to eat each food. Participants’ own food pickiness was related to their judgments about what they would eat, but not whether foods were appropriate for infants. Parents’ judgments of individual foods were inversely related: The more appropriate they rated each food for an infant, the less they were interested in eating that food. These findings are discussed in terms of potential barriers to engaging in social modeling (i.e., parents demonstrating eating and liking the foods they offer to their infants).

## Introduction

Guidelines from the American Academy of Pediatrics on feeding infants solid foods (1) focus on the process of eating, rather than what to eat. These guidelines highlight physical cues indicating that infants are ready for solids (e.g., able to hold their head up, open their mouth when food approaches, move food from a spoon into their throat, have doubled their birth weight) and a few properties solid foods should have (e.g., soft or pureed foods, fortification with iron and zinc, and single-ingredient foods). Many aspects of these guidelines are designed to avoid infant choking or pinpoint allergic reactions, two critical goals for infant safety while eating. Nonetheless, a huge range of foods adhere to these guidelines, leaving parents with many decisions as they introduce new foods. Parents may feel inundated with choices and information. As guidelines note, parents may feel “confused because you have received too much advice from family and friends with different opinions” (1). Indeed, qualitative studies highlight messages from relatives and friends as key sources of information about infant feeding, but also as sources of conflict or stress (2–6).

Given the sparse information from pediatric guidelines in the United States about what foods to select, what are the differences in opinions that the guidelines reference and how might family and friends come to form these different opinions? One potential source of these opinions may be pre-existing concepts, knowledge, or assumptions about what foods are appropriate in a particular context. In a recent study of American adults’ judgments about breakfast foods, rigid thinking about what foods are appropriate for breakfast was observed (e.g., orange juice and cereal were considered more appropriate for breakfast than chili or lamb chops), even if other foods might be more nutritious (7). These patterns can be observed early in life, with American 4- and 5-year-old children making similar judgments as adults about breakfast foods (8), and American 5-year-olds negatively judging people who ate unusual food combinations (9). However, these studies focus on people’s own food preferences or assessments of the food choices of adults, rather than considering parents’ role in selecting foods on behalf of their infants, another ecologically important context.

In addition to these experimental studies, several qualitative studies have examined parents’ beliefs about feeding. Many studies have focused on mothers, as mothers are still primarily responsible for infant feeding and decisions about feeding (3, 4). Similar to pediatric guidelines, a key theme emerging from qualitative studies concerns infants’ ability to eat solid foods (2, 4). Additional parent considerations include whether infants would get enough nutrition from breastmilk/formula alone (6), helping infants sleep (4–6), and resources needed to prepare foods (2). In one study that referenced specific foods to offer, a qualitative study of Latino parents in Northern California referred to traditional practices to select infants’ first solid foods, with chicken soup with vegetables mentioned as the earliest food offered (10). Although several studies refer to infants’ food preferences as an important consideration (2, 3), parents’ own preferences for the foods were not discussed. One study referenced snacks as a way for parents and infants to share foods, but did not directly refer to parents’ own food preferences (6). Nonetheless, parents’ food preferences may influence what foods they choose for infants in important and understudied ways.

The present study examines parents’ judgments of what foods are appropriate for infants, and whether those judgments vary based on participants’ own food preferences. Parents of young children were recruited from Prolific and asked to rate foods offered to infants at different ages (6, 9, and 12 months) and a control group of adult dinner foods based on how appropriate those foods are for infants and how much participants would like to eat those foods. Participants also completed the Food Fussiness subscale of the Adult Eating Behavior Questionnaire (11) as a measure of their general food pickiness.

## Method

### Participants

Participants included adults on Prolific (age range = 21–50 years; 59% reported gender as female, 39% reported male, 2% reported something else) who reported that their youngest child was born from 2019 to 2021 (to ensure that participants recently had a child in the 6–12-month range). One hundred people completed the study. All participants completed at least 70% of the test questions and on average completed 99.84% of questions. One participant was excluded for selecting “no” when asked if they were a parent. See Table 1 for sample demographics.

**TABLE 1:** Sample demographics (*N* and % or mean and SD).

Variable	N (%) or mean (SD)
Age	32.83 (5.78)
**Gender**	
Female	59 (59%)
Male	39 (39%)
Something else/not reported	2 (2%)
**Race/ethnicity**	
White, not Latinx	77 (77%)
Black, not Latinx	7 (7%)
Latinx, any race	7 (7%)
Multiracial, not Latinx	3 (3%)
Asian, not Latinx	5 (5%)
Not reported	1 (1%)
**Income**	
Less than $15,000	5 (5%)
15,000–$25,000	3 (3%)
25,000–$40,000	11 (11%)
40,000–$60,000	16 (16%)
60,000–$90,000	23 (23%)
90,000–$120,000	20 (20%)
More than $120,000	21 (21%)
Not reported	1 (1%)
**Child age (years)**	
All children	4.35 (4.12)
Youngest child	1.61 (0.98)

*N* = 99.

### Materials and procedure

Participants completed a Qualtrics survey in which they were asked to rate a set of 80 foods based on appropriateness for infants and their own preferences. Participants were told, “You will see descriptions of foods that someone might or might not feed to a baby. Imagine a baby that is eating solid foods and is 6- to 12-months old. For each description, we want you to provide two ratings: First, do you think the food is a good food to feed to a baby? The more appropriate and typical you think this food is for a baby, the higher the rating you should provide. Second, would you like to eat this food yourself? The more interested you are in eating the food (now as an adult) exactly as it is described, the higher the rating you should provide.”

From a corpus of 805 foods from observations of mothers offering familiar and unfamiliar foods to their infants at 6, 9, and 12 months (DeJesus et al., in preparation), 10 familiar and 10 unfamiliar foods were randomly selected from each infant age (60 total). In that study, mothers completed a questionnaire about the food they offered in each feeding, including an open-ended question: “What food did you offer your baby during this feeding? Please provide as much detail as possible.” Written descriptions of the foods from that question were cleaned to display similar units (e.g., “ounces” and “oz” were standardized to “ounces”) and formatting (e.g., “Banana—Fresh” was converted to “Fresh banana”). Participants were also shown a control group of 20 adult foods compiled by surveying lab members on what they ate for dinner that week. The purpose of this control group was to assess whether participants would all rate foods as appropriate for infants and/or undesirable to eat, regardless of the actual description. For all foods, participants were only given written descriptions of the foods, without information about the food’s familiarity, the age the food was offered to, or any other descriptors beyond what was provided by mothers in the original study. Foods were displayed in random order. Full text of food descriptions and counts of missing data per item are available on the Open Science Framework (OSF): https://osf.io/etq9y/. Examining missing data per food item, <1% of items were missing for familiar foods, <1% of items were missing for unfamiliar foods, and <1% of items were missing for control foods.

Participants rated the appropriateness and their liking of each food on a 1–5 scale: (1) “not at all,” (2) “slightly,” (3) “moderately,” (4) “very,” and (5) “extremely.” Participants were told, “For both questions, the lowest rating is ‘not at all’ (not at all good for a baby or not at all something you would like to eat) and the highest rating is ‘extremely’ (extremely good for a baby or something you would be extremely happy to eat).” This question format was selected based on a pilot study (reported in supplemental materials on OSF) in which participants were asked to rate foods on a 0–100 scale, but a high rate of incomplete responses was observed and participants tended to use the ends of the scale the most often and used the 25–75 range less often.

Participants then completed the Food Fussiness subscale of the Adult Eating Behavior Questionnaire (11): (1) I often decide that I don’t like a food, before tasting it; (2) I refuse new foods at first; (3) I enjoy tasting new foods; (4) I am interested in tasting new food I haven’t tasted before; and (5) I enjoy a wide variety of foods. Each question had the following response options: (1) Strongly disagree, (2) disagree, (3) neither agree nor disagree, (4) agree, and (5) strongly agree. Scores were averaged with higher scores indicating more pickiness (questions 3, 4, and 5 were reverse coded). Participants then completed a demographic questionnaire.

### Data analysis plan

First, parents’ control food ratings were compared to their infant food ratings. For each question (appropriateness and preference), a mixed-model linear regression (controlling for multiple responses per parent) was performed, with food type (familiar, unfamiliar, and control) as a predictor of parents’ ratings. This was a confirmatory analysis in which we anticipated familiar foods would be rated as most appropriate and control foods rated as highest in terms of participants’ own preferences (but did not have specific predictions about all pairs significantly differing from one another).

Parents’ judgments about the infant foods were then examined. For each question (appropriate, preference), a mixed-model linear regression was performed, with food type (familiar, unfamiliar), infant age (6, 9, and 12 months), and parent food pickiness as predictors of their ratings. Food type again was confirmatory (as we anticipated that familiar foods would be rated as more appropriate), but other variables were exploratory, as we did not have strong expectations about effects of infant age or parent pickiness within infant foods.

To examine associations between parents’ two ratings for each food (appropriateness vs. preference), a mixed-model linear regression was performed, with parents’ own preferences as a predictor of their appropriateness ratings; the model was repeated for individual food types (familiar, unfamiliar, control). This was an exploratory analysis, as we did not have strong expectations regarding the association between questions (i.e., if preference ratings were generally low, there might be no association between preference ratings and appropriateness ratings).

**FIGURE 1:**
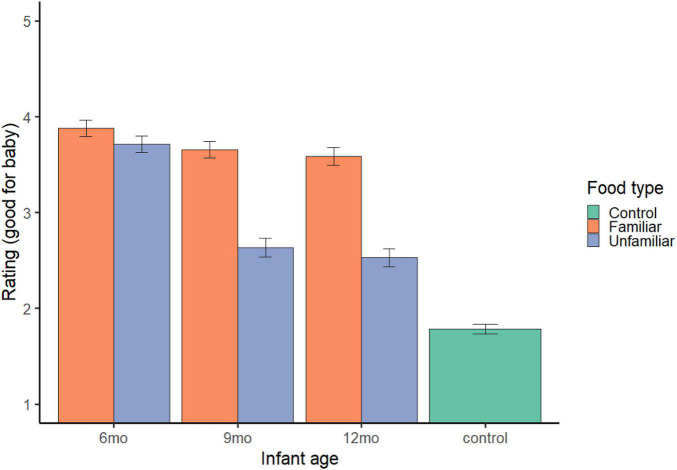
Appropriate and preference judgments. Error bars represent standard error.

**FIGURE 2:**
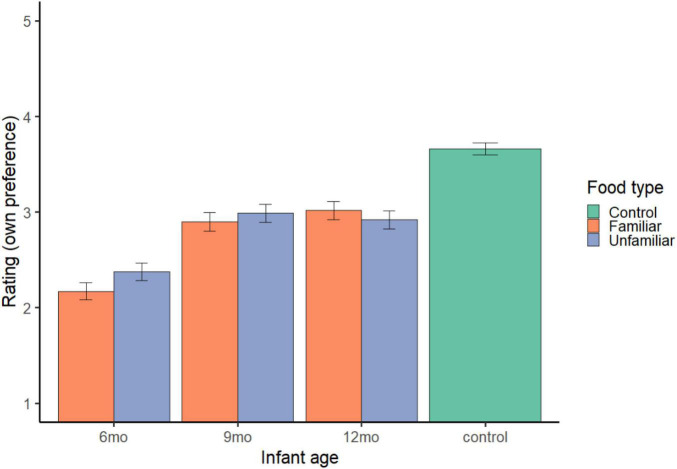
Own preference ratings. Error bars represent standard error.

**FIGURE 3:**
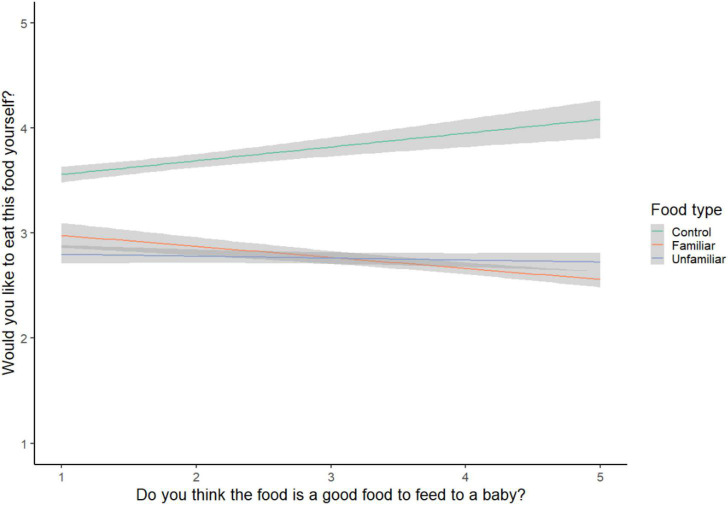
Appropriate vs. preference ratings.

For each model, we report the conditional and marginal R^2^ as indices of model fit (12, 13). See Figures 1–3 for data visualizations and OSF for full regression tables, additional visualizations, deidentified data, and analysis code: https://osf.io/etq9y/. In addition to the pilot study, supplemental analyses include analyses using just the neophobia-related questions of the AEBQ FF and analyses of just participants identifying as female to examine potential gender effects. For both, we observed similar results to the analyses that follow with the full sample and full AEBQ FF subscale.

## Results

### Appropriateness for infant

#### Infant vs. control foods

Parents rated familiar foods (*M* = 3.71, 95% *CI* = 3.66, 3.76; *b* = 1.93, *SE* = 0.04, *t* = 50.81, *p* < 0.001) and unfamiliar foods (*M* = 2.96, 95% *CI* = 2.90, 3.01; *b* = 1.17, *SE* = 0.04, *t* = 30.96, *p* < 0.001) as more appropriate for infants than control foods (*M* = 1.78, 95% *CI* = 1.73, 1.84); model R^2^_*c*_ = 0.35, R^2^_*m*_ = 0.21 (see Figure 1).

#### Familiar vs. unfamiliar infant foods

Food type and infant age predicted parents’ appropriateness ratings (model R^2^_*c*_ = 0.25, R^2^_*m*_ = 0.10). Parents rated familiar foods (*M* = 3.71, 95% *CI* = 3.66, 3.76) as more appropriate than unfamiliar foods (*M* = 2.96, 95% *CI* = 2.90, 3.01; *b* = −0.75, *SE* = 0.04, *t* = −21.39, *p* < 0.001). Parents also rated foods for 6-month-olds (*M* = 3.80, 95% *CI* = 3.74, 3.86; *b* = 0.74, *SE* = 0.04, *t* = 17.19, *p* < 0.001) and 9-month-olds (*M* = 3.14, 95% *CI* = 3.08, 3.21; *b* = 0.09, *SE* = 0.04, *t* = 2.01, *p* = 0.044) as more appropriate than foods for 12-month-olds (*M* = 3.06, 95% *CI* = 2.99, 3.13). Parents’ own pickiness ratings were not associated with their appropriateness judgments (*b* = −0.002, *SE* = 0.06, *t* = −0.03, *p* = 0.978) (see Figure 1).

### Parents’ own preferences

#### Infant vs. control foods

Parents rated familiar foods (*M* = 2.70, 95% *CI* = 2.64, 2.75; *b* = −0.96, *SE* = 0.04, *t* = −23.91, *p* < 0.001) and unfamiliar foods (*M* = 2.76, 95% *CI* = 2.71, 2.82; *b* = −0.90, *SE* = 0.04, *t* = −22.25, *p* < 0.001) as less desirable than control foods (*M* = 3.66, 95% *CI* = 3.60, 3.72); model R^2^_*c*_ = 0.22, R^2^_*m*_ = 0.07 (see Figure 2).

#### Familiar vs. unfamiliar infant foods

Infant age and parent pickiness predicted preference ratings (model R^2^_*c*_ = 0.20, R^2^_*m*_ = 0.06). Parents rated foods for 6-month-olds (*M* = 2.27, 95% *CI* = 2.21, 2.34; *b* = −0.69, *SE* = 0.04 *t* = −15.86, *p* < 0.001) as less desirable than foods for 12-month-olds (*M* = 2.97, 95% *CI* = 2.90, 3.03); foods for 9-month-olds (*M* = 2.94, 95% *CI* = 2.87, 3.01; *b* = −0.02, *SE* = 0.04, *t* = −0.57, *p* = 0.569) did not differ. Parent pickiness was negatively associated with their preference ratings (*b* = −0.21, *SE* = 0.06, *t* = −3.43, *p* < 0.001): The pickier the parent, the less they wanted to eat the described foods. Parent preference ratings did not differ by food type (familiar: *M* = 2.70, 95% *CI* = 2.64, 2.75; unfamiliar: *M* = 2.76, 95% *CI* = 2.71, 2.82; *b* = 0.07, *SE* = 0.04, *t* = 1.85, *p* = 0.064) (Figure 2, right).

### Associations between ratings

Parents’ preference ratings were negatively associated with their appropriateness ratings for each food, *b* = −0.21, *SE* = 0.01, *t* = −18.52, *p* < 0.001, meaning the more parents reported they would eat a food, the less appropriate they rated that food for infants (model R^2^_*c*_ = 0.20, R^2^_*m*_ = 0.04). This association held for familiar foods (*b* = −0.15, *SE* = 0.02, *t* = −8.76, *p* < 0.001, model R^2^_*c*_ = 0.19, R^2^_*m*_ = 0.02) and unfamiliar foods (*b* = −0.09, *SE* = 0.02, *t* = −4.76, *p* < 0.001, model R^2^_*c*_ = 0.17, R^2^_*m*_ < 0.01), but was reversed for control foods (*b* = 0.09, *SE* = 0.02, *t* = 5.51, *p* < 0.001, model R^2^_*c*_ = 0.49, R^2^_*m*_ = 0.01) (see Figure 3).

## Discussion

This study demonstrates associations between parents’ food preferences and whether they view those foods as appropriate for infants. When presented with familiar and unfamiliar foods offered to infants at 6-, 9-, and 12-months and adult dinner control foods, participants rated the infant-directed foods as more appropriate for infants and less likeable compared to control foods, even though participants only had written descriptions of the foods (not who ate the food). Participants’ own food pickiness was negatively associated with their willingness to eat the infant foods, but not their infant appropriateness ratings. Parents also showed an inverse relationship between their appropriateness ratings and their own liking; the more appropriate they rated each food for infants, the less they wanted to eat it themselves. This study makes an important contribution to the study of food cognition by demonstrating systematic associations (as opposed to random responding) between features of foods (whether another parent identified the food as familiar vs. unfamiliar for their infant and what at what age it was offered) and parents’ judgments about those foods, just from written descriptions. In the absence of any sensory information about the foods (i.e., parents could not directly smell or taste the food or see the food’s texture or color), participants still made systematic judgments about whether foods were appropriate for infants.

Another important contribution of this study to the field of food cognition is the finding that parents’ appropriateness and preference judgments regarding infant foods were inversely related: The more appropriate parents rated a food, the less they personally wanted to eat it. This finding highlights potential challenges for employing social modeling to improve early food acceptance. Research on infant social learning highlights that attention to social partners, especially their communicative facial expressions, gestures, and vocalizations [e.g., (14–18)], is important for learning, including in food contexts (19–21). Therefore, social modeling may provide an important mechanism for infants to learn what foods are safe, healthy, and culturally appropriate [see (22)]. Social modeling is recommended to parents of toddlers and children (23–25), but may be limited in infancy if parents avoid eating foods they consider appropriate for infants [see (22)]. Indeed, in an observational study of infant solid food feedings at 6, 9, and 12 months (which provided the food descriptions here), spontaneous social modeling was rare (DeJesus et al., in preparation).

This study has important limitations to address in future research. First, participants only viewed written descriptions of the foods. Written descriptions may or may not convey information about food texture, which infant solid food feeding guidelines discuss in detail, or other sensory properties (e.g., taste, smell, and color). Second, parents were not asked to provide information about their feeding practices. A few parents made substantive comments at the end of the study (*n* = 10), including aspects of their feeding practices or judgments about infant foods, such as “As soon as they started eating solid food, we fed both of our kids everything we ate, just modified for appropriate sizes, spice level and safety,” “I considered the sugar/sodium content for many of the decisions,” and “There were a couple things that I wouldn’t feed to a baby purely off of choking hazard.” However, with a small sample of explanations, systematic conclusions cannot be drawn. Parents were also not asked about their infants’ reactions to solid foods, which may shape parents’ views about what foods are appropriate for infants. Future studies would benefit from interviews with parents about their feeding practices and their infants’ food reactions. Finally, directly asking parents what is appropriate for a baby could be liable to self-presentation or social desirability biases, as parents are very sensitive to the link between feeding choices and perceptions of good parenting [e.g., (3, 4, 26)]. Parents could also be influenced by the description of what it meant for a food to be “good for a baby” (i.e., more appropriate and typical). Asking parents to report separately on specific aspects of this idea, such as appropriateness, typicality, health properties, and infant liking, could yield more nuanced findings. Converging evidence from more indirect or implicit methods would be valuable to provide further insight into parents’ judgments about infant foods.

The present study contributes to a growing body of research on infant feeding practices. Qualitative studies, in which parents (particularly mothers) were interviewed about their judgments about feeding highlight several challenges, including competing information and social comparison with friends and family (2–4, 6), and successful feeding as a part of participants’ identity as mother (4). If successful feeding is central to one’s feeling of competency as a mother, then choosing appropriate foods may feel like a high stakes process, particularly in a confusing information landscape, in which official guidance is sparse but unofficial guidance (e.g., from family members, friends, and social media) may be prominent. Future research is needed to reduce the stress that may result from this confluence of factors.

## Data availability statement

The datasets presented in this study can be found in online repositories. The names of the repository/repositories and accession number(s) can be found below: https://osf.io/etq9y/.

## Ethics statement

The studies involving human participants were reviewed and approved by the UNC Greensboro. The patients/participants provided their written informed consent to participate in this study.

## Author contributions

JD designed the study, analyzed the data, and wrote the manuscript.

## References

[1] American Academy of Pediatrics. *Infant Food and Feeding.* Itasca, IL: American Academy of Pediatrics (2021).

[2] Boak R Virgo−Milton M Hoare A Silva A Gibbs L Gold L Choosing foods for infants: a qualitative study of the factors that influence mothers. *Child Care Health Dev.* (2016) 42:359–69. 10.1111/cch.12323 26935767

[3] Dattilo AM Carvalho RS Feferbaum R Forsyth S Zhao A. Hidden realities of infant feeding: systematic review of qualitative findings from parents. *Behav Sci.* (2020) 10:83. 10.3390/bs10050083 32349324 PMC7287829

[4] Harrison M Brodribb W Hepworth J. A qualitative systematic review of maternal infant feeding practices in transitioning from milk feeds to family foods: maternal infant feeding practices. *Mat Child Nutr.* (2017) 13:e12360. 10.1111/mcn.12360 27696658 PMC6865989

[5] Horodynski M Olson B Arndt MJ Brophy-Herb H Shirer K Shemanski R. Low-income mothers’ decisions regarding when and why to introduce solid foods to their infants: influencing factors. *J Commun Health Nurs.* (2007) 24:101–18. 10.1080/07370010701316247 17563282

[6] Redsell SA Atkinson P Nathan D Siriwardena AN Swift JA Glazebrook C. Parents’ beliefs about appropriate infant size, growth and feeding behaviour: implications for the prevention of childhood obesity. *BMC Public Health.* (2010) 10:711. 10.1186/1471-2458-10-711 21087482 PMC3000404

[7] Bian L Markman EM. Why do we eat cereal but not lamb chops at breakfast? Investigating americans’ beliefs about breakfast foods. *Appetite.* (2020) 144:104458. 10.1016/j.appet.2019.104458 31526837

[8] Bian L Markman EM. What should we eat for breakfast? American and Chinese children’s prescriptive judgments about breakfast foods. *Cogn. Dev.* (2020) 54:100873. 10.1016/j.cogdev.2020.100873

[9] DeJesus JM Gerdin E Sullivan KR Kinzler KD. Children judge others based on their food choices. *J Exp Child Psychol.* (2019) 179:143–61. 10.1016/j.jecp.2018.10.009 30513416 PMC6311432

[10] Beck AL Hoeft KS Takayama JI Barker JC. Beliefs and practices regarding solid food introduction among Latino parents in Northern California. *Appetite.* (2018) 120:381–7. 10.1016/j.appet.2017.09.023 28951238 PMC5784836

[11] Hunot C Fildes A Croker H Llewellyn CH Wardle J Beeken RJ. Appetitive traits and relationships with BMI in adults: development of the adult eating behaviour questionnaire. *Appetite.* (2016) 105:356–63. 10.1016/j.appet.2016.05.024 27215837 PMC4990060

[12] Meteyard L Davies RAI. Best practice guidance for linear mixed-effects models in psychological science. *J Memory Lang.* (2020) 112:104092. 10.1016/j.jml.2020.104092

[13] Sotirchos ES Fitzgerald KC Crainiceanu CM. Reporting of R 2 statistics for mixed-effects regression models. *JAMA Neurol.* (2019) 76:507. 10.1001/jamaneurol.2018.4720 30715077

[14] Behne T Liszkowski U Carpenter M Tomasello M. Twelve-month-olds’ comprehension and production of pointing: twelve-month-olds comprehend pointing. *Br J Dev Psychol.* (2012) 30:359–75. 10.1111/j.2044-835X.2011.02043.x 22882368

[15] Carpenter M Nagell K Tomasello M Butterworth G Moore C. Social cognition, joint attention, and communicative competence from 9 to 15 months of age. *Monogr Soc Res Child Dev.* (1998) 63:i–vi. 10.2307/1166214 9835078

[16] Csibra G Gergely G. Natural pedagogy. *Trends Cogn Sci.* (2009) 13:148–53. 10.1016/j.tics.2009.01.005 19285912

[17] Mumme DL Fernald A Herrera C. Infants’ responses to facial and vocal emotional signals in a social referencing paradigm. *Child Dev.* (1996) 67:3219–37. 10.1111/j.1467-8624.1996.tb01910.x 9071778

[18] Vaish A Striano T. Is visual reference necessary? Contributions of facial versus vocal cues in 12-month-olds’ social referencing behavior. *Dev Sci.* (2004) 7:261–9. 10.1111/j.1467-7687.2004.00344.x 15595366

[19] Liberman Z Woodward AL Sullivan KR Kinzler KD. Early emerging system for reasoning about the social nature of food. *Proc Natl Acad Sci.USA.* (2016) 113:9480–5. 10.1073/pnas.1605456113 27503878 PMC5003271

[20] Shutts K Kinzler KD McKee CB Spelke ES. Social information guides infants’ selection of foods. *J Cogn Dev.* (2009) 10:1–17. 19809590 10.1080/15248370902966636 PMC2756712

[21] Wertz AE Wynn K. Selective social learning of plant edibility in 6- and 18-month-old infants. *Psychol Sci.* (2014) 25:874–82. 10.1177/0956797613516145 24477965 PMC4345201

[22] DeJesus JM Venkatesh S. Does social modeling increase infants’ willingness to accept unfamiliar foods? *Infancy.* (2022) 27:181–96. 10.1111/infa.12442 34812560

[23] Nicklas T Hayes D. Position of the american dietetic association: nutrition guidance for healthy children ages 2 to 11 years. *J Am Dietet Assoc.* (2008) 108:1038–44. 10.1016/j.jada.2008.04.005 18564454

[24] Ogata BN Hayes D. Position of the academy of nutrition and dietetics: nutrition guidance for healthy children ages 2 to 11 years. *J Acad Nutr Dietet.* (2014) 114:1257–76. 10.1016/j.jand.2014.06.001 25060139

[25] Shelov SP. *Feeding and Nutrition: Your 4- to 5-Year-Old. Caring for your baby and young Child: Birth to age 5.* (2009). Available online at: https://www.healthychildren.org/English/ages-stages/preschool/nutrition-fitness/Pages/Feeding-and-Nutrition-Your-4-to-5-Year-Old.aspx (accessed May 15, 2022).

[26] Moscato EM Machin JE. Mother natural: motivations and associations for consuming natural foods. *Appetite.* (2018) 121:18–28. 10.1016/j.appet.2017.10.031 29080704

